# Unveiling novel insights into the role and characteristics of sperm heat shock protein 70 in indigenous Donggala bulls: implications for molecular-based motility markers

**DOI:** 10.5194/aab-69-239-2026

**Published:** 2026-04-20

**Authors:** Berlin Pandapotan Pardede, Asmarani Kusumawati, Erif Maha Nugraha Setyawan, Yonathan Alvin Maruli Asi Sihotang, Sri Gustari, Yulius Duma, Syahruddin Said, Sari Yanti Hayanti, Tulus Maulana, Hikmayani Iskandar, Bambang Purwantara, Erdogan Memili

**Affiliations:** 1 Faculty of Veterinary Medicine, Universitas Gadjah Mada, Yogyakarta, 55281, Indonesia; 2 Department of Reproduction, Obstetrics and Gynecology, Faculty of Veterinary Medicine, Universitas Gadjah Mada, Yogyakarta, 55281, Indonesia; 3 Faculty of Agriculture, Tadulako University, Palu, Central Sulawesi, 94148, Indonesia; 4 Research Center for Applied Zoology, National Research and Innovation Agency (BRIN), Bogor, West Java, 16911, Indonesia; 5 Research Center for Animal Husbandry, National Research and Innovation Agency (BRIN), Bogor, West Java, 16911, Indonesia; 6 Division of Reproduction and Obstetrics, School of Veterinary Medicine and Biomedical Sciences, IPB University, Bogor, West Java, 16680, Indonesia; 7 Cooperative Agriculture Research Center, College of Agriculture, Food and Natural Resources, Prairie View, A&M University, Prairie View, TX 77446, United States

## Abstract

Donggala cattle (*Bos indicus*), an indigenous Indonesian breed, possess strong adaptability to tropical stressors and favourable reproductive traits, making them a strategic focus for genetic improvement via artificial insemination (AI). However, optimizing AI success is constrained by variable semen quality following cryopreservation. This research examined the relationship between the abundance of heat shock protein 70 (HSP70) at both the mRNA and protein levels and post-thaw sperm quality, aiming to assess its viability as a molecular marker of motility in Donggala bulls. A total of six Donggala bulls were included in this study, representing the entire available population at the study site. Based on progressive motility evaluated using computer-assisted sperm analysis (CASA), the bulls were classified into two groups: good motility and poor motility. Comprehensive semen quality assessments were conducted, including viability, plasma membrane and acrosome integrity, DNA fragmentation, and protamine deficiency. HSP70 abundance at both the transcript (RT-qPCR) and protein (enzyme immunoassay) levels was quantified in cryopreserved sperm. Bulls in the good-motility group demonstrated significantly higher values for progressive and total motility, viability, plasma membrane and acrosome integrity, and DNA integrity ( P<0.05 ), as well as elevated HSP70 mRNA and protein abundance. Correlation analyses revealed strong positive associations between HSP70 expression and key sperm quality parameters, particularly progressive motility ( $R^{2}>0.86$ ) and acrosome integrity ( $R^{2}>0.67$ ). These findings suggest that HSP70 enhances sperm resilience to cryopreservation-induced oxidative stress by stabilizing membranes, proteins, and DNA. This study is the first to characterize HSP70 abundance in Donggala bulls, providing foundational evidence of its utility as a motility biomarker. Incorporating HSP70 profiling into sire selection may improve AI outcomes and support the sustainable propagation of this valuable native breed.

## 1 Introduction

Expanding beef cattle populations, particularly in developing countries, is a strategic government initiative to meet domestic demand for animal protein. The preference for local cattle breeds is rooted in their superior adaptability to harsh environmental conditions, which contributes to favourable reproductive traits (Baharun et al., 2025). These traits include consistent annual calving via natural mating, prolonged reproductive lifespan (with females capable of producing up to 10 offspring), and extended mating ability in males, which can remain fertile and mate naturally for up to 20 years (Baharun et al., 2025). Donggala cattle (*Bos indicus*), a prominent indigenous breed in Central Sulawesi, Indonesia, exhibit phenotypic similarities to Ongole-grade cattle and demonstrate notable resilience to drought, heat stress, and parasitic diseases (Baharun et al., 2024). Implementing artificial insemination (AI) programmes is expected to accelerate the propagation of Donggala cattle, thereby increasing the availability of healthy, affordable beef.

However, achieving high success rates in AI remains a considerable challenge. Among the factors influencing AI outcomes, semen quality from breeding bulls is of paramount importance (Tanga et al., 2021). Semen used in AI must contain viable, motile, normal sperm with intact plasma membranes, acrosomes, and unfragmented DNA to ensure successful fertilization and embryo development (Pardede et al., 2020b). Although conventional semen quality parameters, such as progressive motility above 40 %, are often used as minimum thresholds, they cannot reliably predict male fertility (Pardede et al., 2022). Previous research has highlighted the critical role of intrinsic molecular elements in sperm, including specific mRNA transcripts and proteins, in regulating key fertilization processes (Pardede et al., 2020a; Indriastuti et al., 2022). Various mRNA transcripts and protein molecules play crucial roles in regulating normal sperm function, particularly during the fertilization process (Pardede et al., 2020a; Indriastuti et al., 2022). Several of these molecules have been reported as potential biomarkers for evaluating sperm fertility and motility (Rosyada et al., 2023; Pardede et al., 2023, 2024, 2025b; Fatmila et al., 2024; Agil et al., 2025). As previously mentioned, sperm motility remains a globally recognized standard and a critical threshold parameter for determining semen suitability in an AI programme. Among the candidate markers, transition nuclear proteins (TNPs) (Pardede et al., 2024) and A-kinase anchor protein 4 (proAKAP4) (Pardede et al., 2025b) have shown promising potential in assessing sperm motility. Additionally, members of the heat shock protein (HSP) family have been extensively studied for their role in regulating sperm motility and maintaining overall functional integrity (Fatmila et al., 2024; Pardede et al., 2023; Rosyada et al., 2023).

HSP70, a 70 kDa molecular chaperone and a prominent member of the HSP family, has attracted considerable interest due to its diverse roles in male reproductive function (Zhang et al., 2022). It is notably upregulated under cellular stress and is expressed in various stages of sperm development, including spermatogonia, spermatids, and mature sperm (Zou et al., 2019). The expression and localization of HSP70 are dynamic throughout spermatogenesis and persist after ejaculation and cryopreservation (Zhang et al., 2022). Functionally, HSP70 contributes to key processes, including meiosis, sperm maturation, apoptosis inhibition, immune modulation, and sperm-egg membrane fusion (Rosyada et al., 2022). Additionally, it enhances the fluidity of the sperm membrane, supporting sperm survival and function within the female reproductive tract (Zhang et al., 2022). Accumulating evidence has linked HSP70 expression to male fertility parameters. (Fatmila et al., 2024; Pardede et al., 2023; Rosyada et al., 2023). For example, Agarwal et al. (2020) highlighted its involvement in sperm motility, capacitation, and oocyte binding. HSP70 has been detected in the sperm of several cattle breeds, including Friesian Holstein, Tharparkar, and Simmental (Somashekar et al., 2017; Rajoriya et al., 2014; Fatmila et al., 2024; Pardede et al., 2023; Rosyada et al., 2023). Further, Bashiri et al. (2021) demonstrated that HSP70 mRNA in sperm contributes to early embryo development and that dysregulated expression can negatively impact fertility and lead to pregnancy failure. In local Indonesian breeds such as Bali and Madura cattle, increased HSP70 expression at both the mRNA and protein levels has been positively associated with improved semen quality and higher fertility outcomes (Fatmila et al., 2024; Pardede et al., 2023; Rosyada et al., 2023). As Donggala cattle remain one of Indonesia's less-studied native breeds, a detailed molecular investigation of HSP70 expression in this population is warranted. Such profiling could support the identification of genetically superior bulls with favourable sperm traits, thereby enhancing the efficiency of AI programmes and promoting sustainable breeding practices. The present study strengthens the evidence for HSP70's utility and highlights its promise as a molecular biomarker for assessing bull fertility, particularly through its association with sperm motility.

## 2 Materials and methods

### 2.1 Experimental design

The samples used in this study were commercially available cryopreserved semen from Donggala bulls, provided by the Sidera Livestock Breeding Unit in Central Sulawesi, Indonesia. As the research involved only processed semen, no live animals were directly involved in the experimental procedures. Semen collection and cryopreservation were conducted using standardized procedures for all productive-age (5–7 years) Donggala bulls. These procedures, including semen processing, storage, and animal care, adhered to the operational protocols of the Livestock Breeding Unit, were overseen by a licensed veterinarian, and complied with established animal welfare standards. Considering the fact that the bulls in this study are a local breed recently introduced into the region, the breeding centre currently houses a limited population of six individuals, all of which were included in the subsequent analyses. As progressive sperm motility is a critical indicator of semen quality, bull classification in this study was based on post-thaw sperm motility parameters, as reported by Pardede et al. (2024). Thawing was performed by incubating cryopreserved semen straws in a water bath at 37 °C for 30 s. Post-thaw progressive motility was assessed using computer-assisted sperm analysis (CASA) with the Sperm Vision™ system (Minitüb, Tiefenbach, Germany). Semen samples were diluted at a ratio of 10 µL semen to 70 µL of diluent medium, following the manufacturer's recommendations. A 10 µL aliquot of the diluted sample was then placed onto a microscope slide, covered with a cover slip, and evaluated under a microscope equipped with a temperature-controlled stage maintained at 38 °C . Sperm motility was assessed using software presets for bovine semen, analysing 50–150 sperm cells across four microscopic fields. Only progressive motility was included in the analysis.

Each bull's post-thaw motility was calculated as the average of repeated measurements. The overall mean post-thaw motility across all samples was 42.49 %, with individual values ranging from 38.31 % to 48.02 %. Bulls were categorized into two groups, namely good and poor motility, based on their post-thaw motility relative to the population average. Bulls with post-thaw motility above the population mean were classified as having good motility, while those below the mean were categorized as having poor motility. The grouping criteria and corresponding motility values are summarized in Table 1.

**Table 1:** Sperm post-thaw motility phenotypes of the Donggala bulls used for further analysis: bulls 1–3 were defined as good motility, and bulls 4–6 were grouped as poor motility.

Bull no.	Motility status	Average	Difference from
		post-thaw	population
		motility (%)	average (%)
1	Good motility	48.02	5.52
2		46.07	3.57
3		44.33	1.83
4	Poor motility	39.28	-3.22
5		39.00	-3.50
6		38.31	-4.19
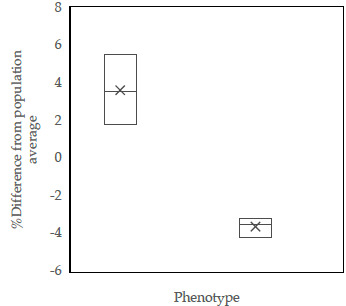			

Bulls were classified as good and poor motility based on average post-thaw motility scores and the percent differences from the population average ( P<0.05 ).

### 2.2 Cryopreserved semen quality assessment

#### 2.2.1 Sperm motility parameter assessment

Sperm motility was analysed using a CASA system. For sperm motility and kinematic analysis, a total of 24 cryopreserved semen straws from these bulls were used, with 4 straws from each bull being analysed as technical replicates. This system was employed not only to evaluate progressive motility but also to quantify total motility and kinematic parameters, including curvilinear velocity (VCL), straight-line velocity (VSL), and average path velocity (VAP). All measurements were performed under standardized settings as detailed in the experimental design. For this analysis, 24 cryopreserved semen straws, comprising samples from all bulls, with three biological replicates per individual, were utilized.

#### 2.2.2 Sperm viability and morphological abnormality assessment

**Figure 1:**
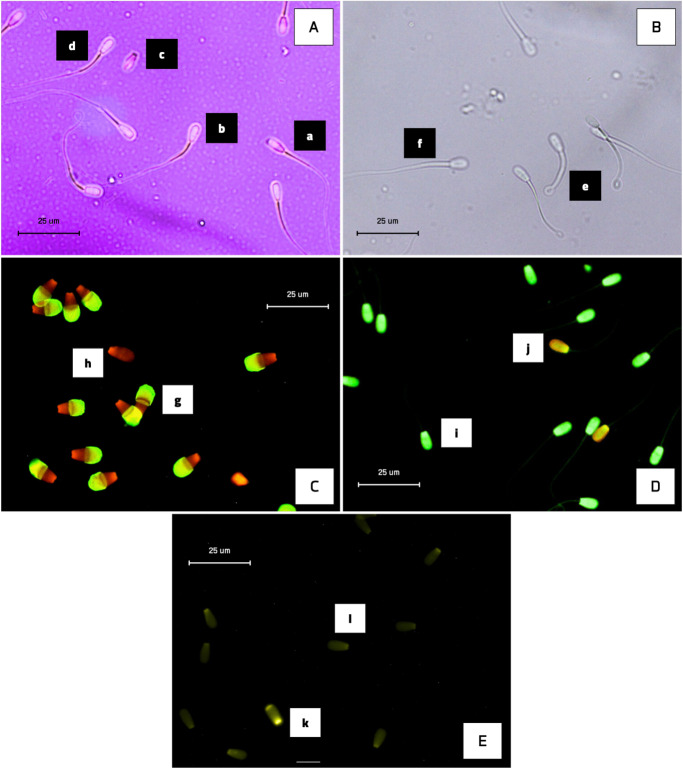
The photomicrograph of Donggala sperm in **(A)** eosine–nigrosine staining, **(B)** HOS test, **(C)** FITC-PNA and PI-based fluorescent staining, **(D)** acridine orange-based fluorescent staining, and **(E)** chromomycin-A3-based fluorescent staining. Non-viable cells exhibited red staining **(A-a)**, while viable sperm appeared to be colourless **(A-b)**. Morphological abnormality is shown in Figure **(A-c)**, and normal sperm are shown in Figure **(A-d)**. HOS-positive (presence of coiled tail) is shown in Figure **(B-e)**, and HOS-negative (absence of coiled tail) is shown in Figure **(B-f)**. Sperm with fluorescent-green acrosomes were categorized as intact acrosomes **(C-g)**, whereas sperm without fluorescence were classified as damaged acrosomes **(C-h)**. Sperm with intact DNA appeared green **(D-i)**, whereas sperm with fragmented DNA appeared as yellow-orange fluorescence **(D-j)**. CMA3 positivity (protamine deficiency) is shown in Figure **(E-k)**, and faint fluorescence **(E-l)** indicates normal protamine content.

Sperm viability and morphology were evaluated using eosine–nigrosine staining. For analysis of sperm viability and morphological abnormalities, 24 cryopreserved semen straws from these bulls were used, with 4 straws per bull being analysed as technical replicates. The tawed semen sample (37 °C for 30 s) was mixed with the stain in equal volumes (10 µL each) and then smeared onto glass slides and dried on a heating stage. Viable sperm remained unstained (colourless), while non-viable sperm took up the eosine dye and appeared red (Fig. 1). For each sample, 250 sperm were assessed using a light microscope at 400× magnification across 10 randomly selected fields (Rosyada et al., 2023).

#### 2.2.3 Sperm plasma membrane integrity assessment

Plasma membrane integrity (PMI) was assessed using the hypo-osmotic swelling test, following the method initially established by Pardede et al. (2025a). For analysis of sperm plasma membrane integrity, a total of 24 cryopreserved semen straws from these bulls were used, with 4 straws from each bull being analysed as technical replicates. After thawing, semen samples were incubated in hypo-osmotic swelling (HOS) solution at 37 °C for 30 min. Following incubation, 5 µL of the treated sample was mounted onto a slide, and 250 sperm were evaluated under a phase-contrast microscope at 400× magnification. Sperm showing tail curling were identified as HOS-positive, signifying intact plasma membranes, whereas those with straight, uncoiled tails were considered HOS-negative (Fig. 1).

#### 2.2.4 Acrosomal integrity assessment

Acrosome membrane integrity was evaluated using fluorescein isothiocyanate-labelled peanut agglutinin (FITC-PNA) in combination with propidium iodide (PI), as described by Pardede et al. (2023). For acrosomal integrity analysis of sperm from these bulls, 24 cryopreserved semen straws were used, with 4 straws per bull being used as technical replicates. Thawed semen samples were smeared onto clean glass slides, air-dried, and then fixed in 96 % ethanol for 10 min. The slides were subsequently incubated with 30 µL of FITC-PNA solution (100 $µg\;{\mathrm{mL}}^{-1}$ ) at 37 °C for 30 min. After incubation, 5 µL of PI (1 $µg\;µL^{-1}$ ) was applied for 5 min. The slides were then rinsed with phosphate-buffered saline (PBS), mounted with coverslips, and observed using a fluorescence microscope (excitation wavelength: 380–420 nm). Sperm displaying green fluorescence in the acrosomal region were considered to have intact acrosomes, whereas red fluorescence indicated acrosomal membrane disruption (Fig. 1). For each specimen, 250 sperm cells were evaluated. All procedures were carried out under dim light to preserve fluorochrome stability.

#### 2.2.5 Sperm DNA integrity assessment

Sperm DNA fragmentation was evaluated using acridine orange (AO) staining, following the protocol outlined by Pardede et al. (2021) and Hasbi et al. (2024). For sperm DNA integrity analysis, a total of 24 cryopreserved semen straws from these bulls were used, with 4 straws from each bull being analysed as technical replicates. Semen was smeared onto glass slides, air-dried, and then fixed in Carnoy's fixative for 2 h. The slides were subsequently stained with a freshly prepared AO solution for 5 min in the dark, rinsed with distilled water, and examined under a fluorescence microscope with an excitation at 450–490 nm. Sperm exhibiting green fluorescence were considered to have intact DNA, while yellow-green to orange fluorescence indicated DNA fragmentation (Fig. 1). A total of 250 sperm were evaluated per sample. For the sperm DNA integrity assessment data, the presented results are from normal sperm and not from sperm with DNA damage.

#### 2.2.6 Protamine deficiency assessment

The protamine content was evaluated using chromomycin A3 (CMA3) staining, as described by Kusumawati et al. (2023). For sperm protamine deficiency analysis, a total of 24 cryopreserved semen straws from these bulls were used, with 4 straws from each bull being analysed as technical replicates. Thawed semen samples were spread onto microscope slides and fixed in a chilled methanol: glacial acetic acid mixture ( 3:1 ) for 5 min at 4 °C . The fixed slides were then incubated for 20 min in a CMA3 staining solution. After incubation, the slides were gently rinsed and allowed to air dry. Sperm cells were visualized using a fluorescence microscope. Sperm exhibiting intense green fluorescence were identified as CMA3-positive, indicative of protamine deficiency. In contrast, those with weaker fluorescence were considered to possess normal protamine content (Fig. 1). For each sample, 250 sperm were assessed.

### 2.3 Bovine HSP-70 mRNA and protein abundance assessment

The expression levels of HSP70 in sperm were assessed at both the mRNA and protein levels, as outlined in the protocol by Pardede et al. (2023). HSP70 mRNA levels were quantified using real-time quantitative polymerase chain reaction (RT-qPCR). Eight cryopreserved semen straws per bull were pooled, thawed at 37 °C for 30 s, and centrifuged at 16000×g for 15 min. Sperm pellets were washed three times with PBS to remove cryoprotectants. Total RNA was extracted using TRIzol Reagent (TRI) reagent following the manufacturer's instructions. Cells were lysed in 1 mL of TRI reagent, followed by the addition of 0.1 mL 1-bromo-3-chloropropane (BCP) or 0.2 mL chloroform. After 3–5 min at room temperature, the aqueous phase was collected, precipitated, washed with ethanol, air-dried, and resuspended. RNA concentration and purity were measured using a NanoDrop™ spectrophotometer. Complementary DNA (cDNA) was synthesized using the SensiFAST™ cDNA Synthesis Kit (Bioline^®^, UK; Bio-65054) following the manufacturer's instructions. Around 20 µL of cDNA was used for qPCR, performed with SsoFast™ EvaGreen^®^ Supermix (Bio-Rad, USA). Each 20 µL reaction contained 10 µL Supermix, 1 µL each of gene-specific primers, 2 µL cDNA, and 6 µL nuclease-free water. Primers targeted the housekeeping gene PPIA (XM_001252921.1) and HSP70 (NM_174344.1). Gene expression levels were analysed using the $2^{-\Delta \Delta C_{t}}$ method, with PPIA as the internal control. For protein analysis, HSP70 levels in spermatozoa were quantified using an enzyme-linked immunosorbent assay (ELISA) kit (cat. no. MBS7606199, MyBioSource.com). Eight semen straws per bull were pooled and tested in duplicate. Samples were thawed at 37 °C , washed with PBS, and centrifuged at 12000×g for 15 min. The sperm lysate (100 µL ) was loaded into enzyme immunoassay (EIA) plate wells and incubated at 37 °C for 90 min. After sequential washes, 100 µL of biotin-conjugated antibody and, later, HRP-streptavidin were added, with intermediate incubations. The signal was developed using 90 µL TMB substrate and was stopped after 10–20 min, and absorbance was read at 450 nm. Protein levels were calculated based on a standard calibration curve.

### 2.4 Statistical analysis

Sperm progressive motility data were assessed using a generalized linear mixed-effect model. The method for categorizing Donggala bulls according to their motility profiles followed previously established protocols. Differences in post-thaw sperm characteristics and in HSP70 mRNA and protein expression levels were examined using an independent-sample t test. All values are reported as the mean ± standard error. Associations among the measured variables were determined through Pearson's correlation analysis. Additionally, linear regression scatterplots were used to explore associations among HSP70 transcript and protein expression, sperm motility, and acrosomal integrity. Statistical computations were conducted using SPSS version 25.0 (IBM Corp., Armonk, NY, USA).

## 3 Results

**Figure 2:**
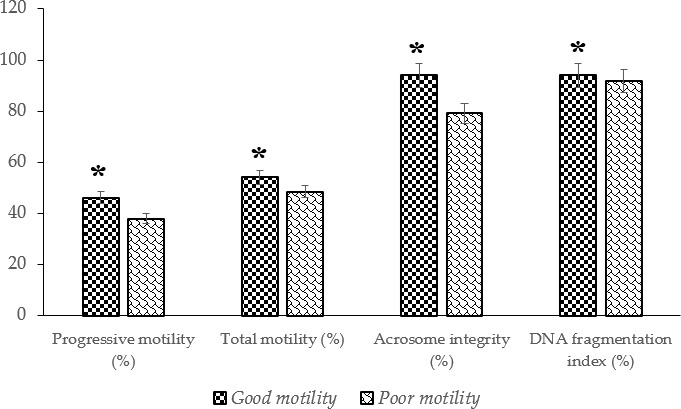
The percentage of progressive and total motility, acrosome integrity, and DNA fragmentation index in Donggala sperm across different motility groups (good vs. poor). The asterisk denotes significant difference when compared to poor motility ( p<0.05 ).

The analysis revealed significant differences ( P<0.05 ) in several semen quality parameters in Donggala bulls, indicating that the cryopreserved semen quality of bulls in the poor-motility group was significantly lower ( P<0.05 ) than that of bulls in the good-motility group (Table 1; Fig. 2). Notably, the good-motility group exhibited higher values in progressive motility ( 46.13±1.45% vs. 37.93±1.79% ) and total motility ( 54.19±1.84% vs. 48.49±1.90% ), acrosome integrity ( 94.00±0.70% vs. 79.12±1.14% ), DNA fragmentation index ( 94.12±0.76% vs. 91.87±0.50% ), sperm viability ( 56.67±1.63% vs. 50.91±1.69% ), and plasma membrane integrity ( 49.83±1.30% vs. 44.37±1.43% ) ( P<0.05 ) (Fig. 2; Table 2).

**Table 2:** The difference in sperm parameters between motility groups, specifically good vs. poor motility.

Parameters	Good motility	Poor motility
VCL ( $µm\;s^{-1}$ )	$92.35\pm 3.06^{a}$	$88.18\pm 3.73^{a}$
VAP ( $µm\;s^{-1}$ )	$67.99\pm 1.87^{a}$	$65.30\pm 2.31^{a}$
VSL ( $µm\;s^{-1}$ )	$45.19\pm 1.14^{a}$	$44.13\pm 1.78^{a}$
Viability (%)	$56.67\pm 1.63^{a}$	$50.91\pm 1.69^{b}$
PMI (%)	$49.83\pm 1.30^{a}$	$44.37\pm 1.43^{b}$
Abnormality (%)	$6.37\pm 0.51^{a}$	$14.12\pm 5.55^{a}$
Protamine deficiency (%)	$1.17\pm 0.40^{a}$	$1.21\pm 0.49^{a}$

VCL: curvilinear velocity; VAP: average path velocity; VSL: straight-line velocity; PMI: plasma membrane integrity. The letters (a, b) in each motility group indicate a significant difference ( p<0.05 ).

In contrast, no significant differences ( P>0.05 ) were observed between the groups for other parameters, including kinematic parameters, namely curvilinear velocity (VCL: $92.35\pm 3.06\;µm\;s^{-1}$ vs. $88.18\pm 3.73\;µm\;s^{-1}$ ), average path velocity (VAP: $67.99\pm 1.87\;µm\;s^{-1}$ vs. $65.30\pm 2.31\;µm\;s^{-1}$ ), and straight-line velocity (VSL: $45.19\pm 1.14\;µm\;s^{-1}$ vs. $44.13\pm 1.78\;µm\;s^{-1}$ ), as well as sperm morphological abnormalities ( 6.37±0.51% vs. 14.12±5.55% ) and protamine deficiency ( 1.17±0.40% vs. 1.21±0.49% ) (Table 2).

**Figure 3:**
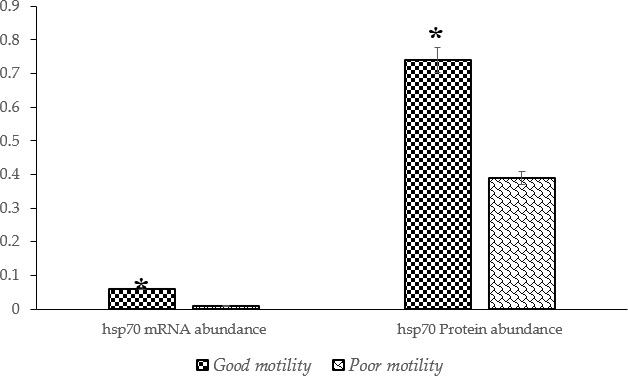
The percentage of HSP70 mRNA and protein abundance in Donggala sperm between good and poor motility. The asterisk denotes significant difference when compared to good motility ( p<0.05 ).

A significantly higher ( P<0.05 ) abundance of HSP70 mRNA and protein was detected in sperm from Donggala bulls in the good-motility group compared to in the poor-motility group (Fig. 3).

These findings were supported by correlation analysis, which revealed a strong positive association between HSP70 mRNA ( P<0.003 ) and protein ( P<0.001 ) abundance and progressive motility (Tables 3 and 4).

**Table 3:** Correlation between HSP70 mRNA expression and semen quality parameters in the Donggala bulls. ^a^

Sperm parameters	Correlation coefficient	P value
HSP70 mRNA vs. HSP70 protein	0.869**	<0.000
HSP70 mRNA vs. progressive motility	0.577**	<0.003
HSP70 mRNA vs. total motility	0.486*	<0.043
HSP70 mRNA vs. VCL	0.242	<0.275
HSP70 mRNA vs. VAP	0.120	<0.254
HSP70 mRNA vs. VSL	0.787	<0.575
HSP70 mRNA vs. sperm viability	0.475*	<0.041
HSP70 mRNA vs. PMI	0.470*	<0.020
HSP70 mRNA vs. sperm abnormality	-0.219	<0.303
HSP70 mRNA vs. AI	0.935**	<0.000
HSP70 mRNA vs. DFI	0.501*	<0.013
HSP70 mRNA vs. Prota-def	-0.039	<0.855

VCL: curvilinear velocity; VAP: average path velocity; VSL: straight-line velocity; PMI: plasma membrane integrity; AI: acrosome integrity; DFI: DNA fragmentation index; Prota-def: protamine deficiency. ^a^ The Pearson correlation represents all values obtained, regardless of the grouping of bulls based on motility. * Correlated significantly at the 0.05 level. ** Correlated significantly at the 0.01 level.

**Table 4:** Correlation between HSP70 protein abundance and semen quality parameters in the Donggala bulls. ^a^

Sperm parameters	Correlation coefficient	P value
HSP70 protein vs. HSP70 mRNA	0.869**	<0.000
HSP70 protein vs. progressive motility	0.615**	<0.001
HSP70 protein vs. total motility	0.495*	<0.014
HSP70 protein vs. VCL	0.167	<0.435
HSP70 protein vs. VAP	0.189	<0.375
HSP70 protein vs. VSL	0.115	<0.591
HSP70 protein vs. sperm viability	0.515**	<0.010
HSP70 protein vs. PMI	0.607**	<0.002
HSP70 protein vs. sperm abnormality	-0.319	<0.129
HSP70 protein vs. AI	0.787**	<0.000
HSP70 mRNA vs. DFI	0.474*	<0.049
HSP70 mRNA vs. Prota-def	0.031	<0.887

VCL: curvilinear velocity; VAP: average path velocity; VSL: straight-line velocity; PMI: plasma membrane integrity; AI: acrosome integrity; DFI: DNA fragmentation index; Prota-def: protamine deficiency. ^a^ The Pearson correlation represents all values obtained, regardless of the grouping of bulls based on motility. * Correlated significantly at the 0.05 level. ** Correlated significantly at the 0.01 level.

Moreover, total motility ( P<0.043 ; P<0.014 ), viability ( P<0.041 ; P<0.010 ), plasma membrane integrity ( P<0.020 ; P<0.002 ), acrosome integrity ( P<0.000 ; P<0.000 ), and DNA fragmentation ( P<0.013 ; P<0.049 ) were also significantly correlated with HSP70 mRNA and protein abundance (Tables 3 and 4).

**Figure 4:**
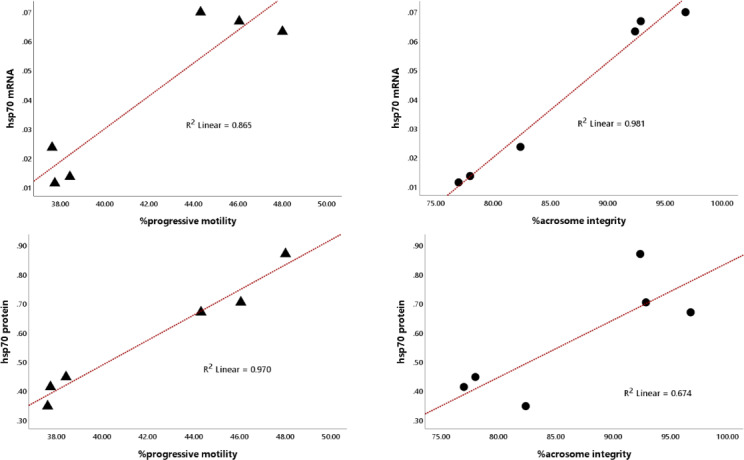
Regression plot of the relationship between HSP70 mRNA and protein abundance with sperm progressive motility and acrosome integrity, regardless of bull grouping based on motility.

Notably, the abundance of HSP70 mRNA and protein in bovine sperm was significantly ( P<0.01 ) associated with progressive motility and acrosome integrity, showing high linearity, with coefficients of determination ( $R^{2}$ ) for HSP70 mRNA of 0.865 and 0.981 and for HSP70 protein of 0.970 and 0.674 (Fig. 4).

## 4 Discussion

Although various studies have investigated the role of HSP70 as a molecular marker to assess fertility and sperm quality in bulls (Fatmila et al., 2024; Pardede et al., 2023; Rajoriya et al., 2014), this study is the first to report findings specifically relating to Donggala bulls. This local Indonesian breed remains understudied, and foundational molecular data, especially on fertility, are limited. Considering the critical role of high-fertility sires in successful breeding programmes, particularly in regions where Donggala cattle are raised for genetic improvement and productivity, identifying reliable fertility markers is imperative. HSP70, known for its involvement in stress response and protein stabilization (Evans et al., 2010), is hypothesized to play a crucial role in regulating sperm quality in this breed. Therefore, the present study aimed to investigate the expression profile of HSP70 at both mRNA and protein levels and its relationship with multiple sperm quality parameters to evaluate its potential as a sperm motility marker for Donggala bulls. To carry out this investigation, bulls housed at a breeding centre were divided into two groups based on their progressive sperm motility, a key indicator used routinely to determine whether frozen semen meets distribution standards for artificial insemination (AI). This categorization reflects practical relevance as progressive motility is commonly applied to select semen doses for field use. Analysis showed that bulls in the good-motility group exhibited significantly greater progressive and total motility than those in the poor-motility group ( P<0.05 ). These findings validate the grouping approach and establish a physiological distinction between the groups.

Further analysis revealed that bulls with good motility also exhibited significantly elevated levels of HSP70 mRNA and protein ( P<0.05 ), suggesting a clear association between this molecular chaperone and sperm motility. It is well documented that the cryopreservation-thawing process induces structural and biochemical stress in sperm cells, often leading to plasma membrane damage, mitochondrial dysfunction, and impaired motility (Hai et al., 2024). These adverse effects are primarily due to the generation of reactive oxygen species (ROS) during freezing and thawing, resulting in oxidative stress (Len et al., 2019). HSP70 plays a pivotal role in counteracting stress-induced changes by regulating cellular enzymatic activity and stabilizing protein folding (Singh et al., 2025). In our study, reduced motility in the poor-motility group coincided with significantly lower HSP70 mRNA and protein abundance. This may reflect compromised antioxidant defence mechanisms, in which reduced HSP70 expression limits the activity of crucial antioxidant enzymes, leading to ROS accumulation and subsequent sperm damage (Liu et al., 2025; Pardede et al., 2023). Supporting this interpretation, HSP70 has been shown to inhibit stress-activated protein kinases, including p38 and JNK, which promote cell apoptosis under stress conditions (Zhang et al., 2020; Pardede et al., 2023). Therefore, low HSP70 levels may allow stress pathways to proceed unchecked, resulting in decreased sperm viability and function (Pardede et al., 2023).

Beyond motility, our study revealed significant differences in sperm viability between the two groups. This is consistent with previous work by Pardede et al. (2023), which suggested that viability is an important predictor of sperm cryotolerance – the ability of sperm to survive the freeze–thaw process. In the present study, HSP70 mRNA and protein levels were positively correlated with sperm viability, indicating that higher HSP70 expression may contribute to improved post-thaw survival. This was further supported by findings on plasma membrane integrity, which was significantly higher in the high-motility group ( P<0.05 ) and correlated with increased HSP70 expression. Heat shock proteins, including HSP70, are a class of molecular chaperones essential for cell survival under stress (Hu et al., 2022). They help maintain protein structure, prevent aggregation of misfolded proteins, and support the refolding of denatured proteins (Rakib et al., 2024). The dynamic expression of HSPs in response to thermal and oxidative stress allows cells to adapt to harsh conditions, such as those experienced during semen cryopreservation (Szyller and Bil-Lula, 2021). In this context, the observed relationship between HSP70 and both viability and membrane integrity suggests that this protein is crucial for sperm resilience during freezing.

Moreover, reduced HSP70 levels may impair membrane fluidity, a critical factor for motility and fertilization capacity (Fatmila et al., 2024). HSP70 has been hypothesized to assist in the folding of membrane proteins and may interact directly with lipid membranes (De Maio and Hightower, 2021). In the current study, poor-quality sperm, characterized by low motility, viability, and membrane integrity, coincided with decreased HSP70 expression, suggesting that the protein was either depleted during stress adaptation or synthesized insufficiently. Disruption of membrane fluidity, potentially caused by abnormal protein folding, can reduce motility, as reported by Marinko et al. (2019). Importantly, a positive correlation between HSP70 abundance and sperm motility supports the idea that enhanced HSP70 synthesis improves cellular resilience. HSP70 may promote ${\mathrm{Ca}}^{2+}$ -ATPase activity, which is critical for maintaining calcium homeostasis and membrane function, and may also activate superoxide dismutase (SOD) to protect against oxidative damage (Liu et al., 2025; Pardede et al., 2023). Under conditions of stress, the phosphorylation state of EIF-2 α (eukaryotic initiation factor-2 alpha) influences HSP expression (Alagar Boopathy et al., 2022). Stress-induced dephosphorylation of EIF-2 α initiates translation of stress-protective proteins such as HSP70 (Hu et al., 2010). However, bulls with poor motility showed reduced HSP70 abundance, suggesting that the sperm may have sustained irreversible cellular damage beyond their stress-coping capacity.

Additionally, HSP70 proteins possess ATPase activity, essential for their chaperone function and the refolding of stress-denatured proteins (Mayer and Bukau, 2005). ATP hydrolysis is required to drive this activity (Mayer and Bukau, 2005). Since sperm motility depends heavily on mitochondrial adenosine triphosphate (ATP) production, reduced ATPase activity, possibly due to cold shock during cryopreservation, could impair motility (Zhang et al., 2024). Although velocity-related parameters, such as VCL, VAP, and VSL, did not differ significantly between groups, the significant differences in progressive and total motility suggest that energy-dependent processes regulated by HSP70 may selectively influence forward motility. Another key finding was the considerable difference in acrosome integrity between the two groups. HSP70 was not only expressed in the cytoplasm. Still, it was also found to be localized in the acrosomal and post-acrosomal regions of sperm, where it is likely to contribute to the stability of membrane-associated proteins during capacitation and the acrosome reaction (Grassi et al., 2022). The acrosome plays a vital role in fertilization by facilitating sperm penetration into the zona pellucida (Hirose et al., 2020). Disruption of the acrosomal structure, potentially caused by oxidative stress and reduced HSP70 levels, can therefore directly compromise fertilizing ability (Castro et al., 2025).

Moreover, sperm DNA integrity was also compromised in the low-motility group. DNA fragmentation is often the result of excessive ROS generation, driven by oxidative stress during cryopreservation (Fleming and Thomson, 2025). ROS can damage membrane lipids, impair protein function, and fragment DNA, ultimately reducing sperm fertilizing capacity (Wang et al., 2025). In this study, bulls with low motility not only had higher DNA damage but also had lower HSP70 expression, further suggesting that this molecular chaperone plays a protective role in maintaining DNA integrity under stress. Overall, the bulls with lower progressive motility consistently exhibited poorer sperm quality, including decreased viability, compromised membrane and acrosome integrity, and increased DNA damage, all of which were significantly associated with lower levels of HSP70 mRNA and protein. These findings suggest that insufficient HSP70 expression impairs sperm's ability to cope with stress during the freeze–thaw process, leading to structural and functional damage. Previous studies have reported HSP90 as a potential biomarker of cryotolerance in bull sperm (Ugur et al., 2019). Our study builds on this knowledge by demonstrating the potential of HSP70, particularly in Donggala bulls, as a marker of sperm motility and overall sperm quality. These findings are especially relevant for AI centres aiming to improve sire selection using molecular fertility indicators. In the future, if specific threshold levels of HSP70 expression associated with fertile or subfertile bulls can be established, this biomarker may serve as an additional criterion for bull selection and fertility evaluation in AI programmes. Although further research is required to clarify the regulatory mechanisms and validate these findings in larger populations, the present study provides foundational insights into the potential application of HSP70 in bull fertility assessment.

## 5 Conclusions

In conclusion, this study highlights the important role of HSP70 in maintaining sperm quality in Donggala bulls under cryopreservation stress. The mRNA and protein levels of HSP70 were positively correlated with progressive motility, viability, membrane and acrosome integrity, and DNA stability. These findings suggest that HSP70 may serve as a complementary molecular biomarker for bull fertility evaluation, particularly for sperm motility, which remains the primary parameter used in breeding centres. Given the very limited population of Donggala bulls, identifying such biomarkers may facilitate future selection and expansion of qualified breeding bulls for artificial insemination programmes while supporting the conservation and sustainable genetic improvement of this local breed.

## Data Availability

The datasets are available upon request from the corresponding author.
